# Health benefits of anthocyanins against age-related diseases

**DOI:** 10.3389/fnut.2025.1618072

**Published:** 2025-06-20

**Authors:** Xiaojie Ma, Zhihai Jin, Zhijian Rao, Lifang Zheng

**Affiliations:** ^1^College of Physical Education, Shanghai University, Shanghai, China; ^2^College of Physical Education, Shanghai Normal University, Shanghai, China; ^3^Exercise Biological Center, China Institute of Sport Science, Beijing, China

**Keywords:** anthocyanidins, age-related diseases, neurodegenerative disease, bone diseases, cardiovascular disease, cancer

## Abstract

Anthocyanins, a class of polyphenol flavonoids widely present in various fruits and vegetables, have attracted significant attention due to their potent anti-inflammatory, antioxidant, and anti-aging properties. Recent studies indicate that anthocyanins may play important roles in extending life and preventing or treating age-related diseases. This review systematically summarizes the chemical characteristics of anthocyanins and their potential roles in age-related diseases, including lifespan extension, neurodegenerative diseases, skeletal diseases, cardiovascular diseases, cancer, and metabolic syndrome. Furthermore, we explore the effects of anthocyanins on age-related diseases and their potential mechanisms of action to establish a theoretical foundation for future clinical applications.

## 1 Introduction

Aging is a natural process where the ability of organisms to adapt both physically and mentally to their environment gradually decreases, ultimately resulting in death. Aging drives the development of various diseases related to old age. Common age-related diseases include cardiovascular disease (CVD) (1), neurodegenerative disease (2), cancer (3), metabolic syndrome (4) and bone diseases (5). These conditions can diminish the quality of life for older adults and impose a significant economic burden on families and society. Thus, creating new and effective anti-aging strategies to reduce or delay age-related diseases and improve the quality of life for older adults is a crucial public health challenge that must be addressed. Strong evidence from both animal and human studies shows a clear but complex link between nutrition and aging (6–8). In recent years, numerous studies have investigated the anti-aging effects of nutritional strategies, such as antioxidant nutrient supplementation, which helps reduce health risks and promote healthy aging.

Vegetables and fruits, abundant in polyphenolic compounds, have been shown to effectively extend lifespan and reduce the risk of age-related diseases (9, 10). Anthocyanins (ACNs), a class of water-soluble plant pigments classified as flavonoids, are abundantly found in numerous fruits and vegetables such as blueberries, blackberries, red grapes, and purple cabbage. ACNs provide vibrant coloration to plants and exhibit various biological properties, such as anti-inflammatory, antioxidant, and antitumor activities. Moreover, the molecular structure of anthocyanins, which includes conjugated cyclic systems and hydroxyl substituents-especially catechol moieties-confers potent antioxidant capacity (11). Recent clinical and experimental studies show that anthocyanins can extend lifespan and help prevent or alleviate various age-related diseases, including neurodegenerative diseases, cardiovascular disorders, metabolic syndrome, bone diseases, and cancer. Therefore, understanding the therapeutic effects and underlying mechanisms of anthocyanins in age-related diseases has significant scientific and clinical implications.

## 2 Chemical properties of anthocyanins

Anthocyanins are important natural pigments found in plants, classified as flavonoids. They have a unique structure known as a benzopyran skeleton, which consists of a benzene ring attached to a pyran ring. The structure of anthocyanins greatly affects their stability, solubility, and bioavailability, which in turn influences their use in food, pharmaceuticals, and nutraceuticals. Currently, over 650 different anthocyanin compounds have been identified in plants. These compounds can be categorized into six main aglycone variants based on their substituent patterns: Pelargonidin (Pg), Cyanidin (Cy), Delphinidin (Dp), Peonidin (Pn), Malvidin (Mv), and Petunidin (Pt) (12). Anthocyanins are mainly found in a variety of fruits, vegetables, and some flowers, such as blueberries, blackberries, red grapes, purple cabbage, and purple sweet potatoes. The chemical structure of anthocyanins contains multiple hydroxyl and carboxyl groups, functional moieties that confer potent antioxidant capacity and bioactivity. Studies, both *in vivo* and *in vitro*, have shown that anthocyanins have various biological functions, including antioxidant, anti-inflammatory, anti-aging, antimicrobial, anti-tumor, hypoglycemic, vision-protective, and immunomodulatory effects (13–15). In addition, more and more studies have shown that anthocyanins have important roles in prolonging life span and the prevention or treatment of aging-related diseases, including cardiovascular diseases (16), neurodegenerative diseases (17), metabolic diseases (18), skeletal diseases (19), cancer (20) and eye-related diseases (21).

## 3 Anthocyanins and lifespan extension

Extending lifespan is a key objective of anti-aging research and serves as a crucial indicator of its effectiveness. One of the mechanisms of aging is the excessive accumulation of oxygen radicals, which leads to oxidative damage. Studies have shown that anthocyanins have antioxidant biological activity and can prolong the life span of Drosophila and *Caenorhabditis elegans* (*C. elegans*). For example, studies indicate that black rice anthocyanins extract (BRAE) can extend Drosophila lifespan by 20% while also delaying the loss of motor function (22). Additionally, Zuo et al. reported an increase in Drosophila lifespan of 14% due to BRAE (23). The proposed mechanism indicates that BRAE may enhance the mRNA levels of CuZnSOD (SOD1), MnSOD (SOD2), catalase (CAT), and Rpn11 in fruit flies, while simultaneously downregulating the mRNA level of methuselah (Mth). This modulation strengthens the antioxidant system and contributes to lifespan extension in fruit flies (23). Honeysuckle (Lonicera pallasii) extract is an excellent source of anthocyanins. Studies have shown that 100 μM honeysuckle extract, by activating the silent information regulator 6 (Sirt 6)/Lelch-like ECH-associated protein 1 (Keap 1)/nuclear factor-erythroid 2-related factor 2 (Nrf2) signaling pathway, can increase the lifespan of *Drosophila melanogaster* by 8%. The integrity of the intestinal barrier increased by 4%; inhibition of Sirt-6 expression blocked the effect of honeysuckle extract on lifespan extension in *Drosophila melanogaster* (24). Furthermore, purple sweet potato extract (PSPE) is not only rich in anthocyanins but also exhibits greater stability compared to anthocyanins found in other plants, such as blueberries and cranberries. Studies have shown that PSPE activates the autophagy pathway by increasing the activity of antioxidant enzymes and inhibiting the mammalian target of rapamycin (mTOR) pathway, improving intestinal homeostasis and mitigating intestinal barrier dysfunction, thus extending the lifespan of Drosophila (25, 26).

Moreover, anthocyanins can also effectively improve the lifespan of *C. elegans*. Studies show that PSPE improves the antioxidant enzyme activity in *C. elegans* and reduces malondialdehyde, reactive oxygen species (ROS), and lipofuscin accumulation. This leads to a 26.7% increase in their lifespan. In contrast, fermented PSPA extends their lifespan by 37.5% (27). The primary component of red cabbage anthocyanins, cyanidin-3-diglycoside-5-glucoside (CY3D5G), exhibits antioxidant activity. The study demonstrated that the derivatives of red cabbage CY3D5G (RCJ) significantly increased the survival rate and average lifespan of *C. elegans* under oxidative and heat stress, with improvements of 171.63, 31.64, and 28.16%, respectively. The life-prolonging effect of RCJ may be related to the heat shock transcription factor pathway, deacetylase signaling pathway and calmodulin kinase II pathway (28). Alternatively, Chen et al. showed that anthocyanin-rich purple wheat has a lifespan-extending effect, partially dependent on the activation of DAF-16/FOXO transcription factors (29). Similarly, the nutrients from mulberry anthocyanin extract (MAE) can effectively prolong the longevity of paraquat-damaged *C. elegans* by inhibiting mitogen-activated protein kinase (MAPK)/Nrf2 signaling *in vitro* (30). Moreover, other natural compounds such as wheat bran, Dendrobium officinale flower, extracts of Tsai Tai, purple pitanga fruit, *lycium barbarum* extracts have been shown to effectively extend the life span, but the specific mechanism needs to be further explored (31–35). In summary, these studies underscore the crucial role of anthocyanins in promoting healthy aging. Anthocyanins play a key role in delaying aging and improving lifespan by activating autophagy, inhibiting oxidative stress, and improving intestinal homeostasis, providing new perspectives for future aging research (Table 1).

**Table 1:** Summary of anthocyanins sources and its anti-aging ability in different models.

Anthocyanins	Experiment model	Dose of application	Key findings
Black rice anthocyanin extract (22)	*D. melanogaster*	5 mg/mL	↑Mean lifespan; ↑Climbing ability.
Black rice anthocyanin extract (23)	*D. melanogaster*	30 mg/mL	↑Mean lifespan; ↑Antioxidant capacity; ↑The mRNA of antioxidant enzyme (SOD1, SOD2, CAT and Rpn11); ↓The mRNA of Mth.
Honeysuckle (Lonicera pallasii) extract (24)	*D. melanogaster*	100 μM	↑Median and maximum lifespan; ↑Integrity of the intestinal barrier; Activate Sirt6/Keap1/Nrf2 signaling pathway.
Purple sweet potato extract (25)	*D. melanogaster*	0.5 mg/mL and 2.0 mg/mL	↑Mean lifespan; ↑Antioxidant capacity; ↓The mRNA of mTOR; ↑The mRNA of autophagy (Atg1, Atg5, Atg8a and Atg8b); Improves intestinal homeostasis.
Purple sweet potato extract (26)	*D. melanogaster*	2.0 mg/mL and 5.0 mg/mL	↑Mean and maximum lifespan; ↑Antioxidant capacity; Activates the autophagy; Improves intestinal homeostasis.
Purple sweet potato extract and fermented purple sweet potato extract (27)	*C. elegans*	80 μg/mL	↑Mean lifespan; ↓The intracellular ROS; ↑The mRNA of longevity-related genes (daf-16, hsp-16.2, sir-2.1, skn-1, and sod-3).
Red cabbage juice (28)	*C. elegans*	1, 2, 3 and 5%	↑Mean lifespan; ↑The survival rate in oxidative and thermal stress; ↑The mRNA (hsp-16.1, hsp-16.2); ↓The mRNA osr-1.
Purple wheat extract (29)	*C. elegans*	10, 50 and 100 μg/mL	↑Mean lifespan; ↑The nuclear localization of DAF-16/FOXO.
*Morus alba* L. extract (30)	*C. elegans*	100 μg/mL	↑The mean life in oxidative stress; ↓The level of MDA and lipofuscin; ↑The DAF-16/FOXO, SKN/Nrf2 and PMK-1/p38 pathways
Dendrobium officinale L. freeze-dried extract (31)	*C. elegans*	150 μL	↑Mean lifespan; ↑The survival rate in oxidative and thermal stress.
Wheat bran extract (32)	*D. melanogaster*	0.1 g/mL	↑Mean lifespan; The survival rate in oxidative stress was not affected; ↓Female survival rate in starvation ↑Female survival rate in thermal stress;
*Brassica chinensis* (Tsai Tai) extract (33)	*C. elegans*	2 mg/mL	↑Mean lifespan; ↑The survival rate in oxidative stress; ↓The intracellular ROS.
*Eugenia uniflora* L. extract (34)	*C. elegans*	5–500 μg/mL	↑The survival rate in oxidative and thermal stress; ↓The intracellular ROS; ↑The nuclear localization of DAF-16.
*Lycium barbarum* berry extract (35)	*C. elegans*	5 mg/mL	The mean lifespan depending on sir-2.1 pathway; ↑Sir-2.1 activity.

↑, increase or enhance; ↓, reduce or inhibit; ROS, reactive oxygen species; SOD1, superoxide dismutase 1; SOD2, superoxide dismutase 2; CAT, catalase; Mth, methuselah; Sirt6, Sirtuin6; Keap1, Kelch-1ike ECH- associated protein; Nrf2, Nuclear factor erythroid 2-related factor 2; FOXO, Forkhead box O; p38, p38 mitogen-activated protein kinase.

## 4 Role of anthocyanin in age-related diseases

### 4.1 Anthocyanins and neurodegenerative diseases

Aging is the primary risk factor for neurodegenerative diseases, particularly Alzheimer’s Disease (AD), Parkinson’s Disease (PD), and Amyotrophic Lateral Sclerosis (ALS), all of which become more prevalent with age (36). Recent studies have shown that anthocyanins and anthocyanin-rich extracts can alleviate the cognitive deficits associated with PD, AD, and ALS.

#### 4.1.1 Anthocyanins and Alzheimer’s disease

Alzheimer’s disease (AD) is a common and severe neurodegenerative disorder related to aging, marked by cognitive decline and synaptic dysfunction. Currently, about 50 million people aged 65 and older have Alzheimer’s disease (AD) worldwide, and this number is expected to triple by 2050 (37). Human studies have shown that consuming 200 milliliters of cherry juice daily for 12 weeks significantly enhances language fluency, short-term memory, and long-term memory in older adults aged 70 and above with mild to moderate dementia (38). Supplementation with anthocyanin-rich blueberry concentrate (30 mL/day for 12 weeks) may not only improve brain perfusion and activation in brain areas associated with cognitive function in healthy older adults (39), but also enhance neural activation in patients with mild cognitive impairment and strength neural responses during working memory challenges in older adults with cognitive decline (40). Animal studies have also shown that anthocyanin-rich blackcurrant extract (3% anthocyanin for 9 weeks) also improves long-term recognition memory and normalized anxiety levels in senescence-accelerated mouse prone 8 (SAMP 8) mice (41). Additionally, mulberry extract (0.18 and 0.9% mulberry extract for 12 weeks) reduces brain *β*-amyloid levels and improves learning and memory in SAMP 8 mice (42). These studies indicate that anthocyanins can effectively address age-related cognitive decline and may serve as a promising compound for preventing and treating Alzheimer’s disease.

The brain is particularly vulnerable to oxidative stress, as previous studies indicate that reactive oxygen species (ROS) levels are significantly elevated in the brains of Alzheimer’s disease (AD) patients and animal models (43). Mechanistic studies on the neuroprotective effects of anthocyanins indicate that Korean black bean anthocyanin (12 mg/kg/day for 30 days) regulates the phosphorylated phosphatidylinositol 3-kinase (p-PI3K)/protein kinase B (Akt)/glycogen synthase kinase 3β (GSK3β) pathway, thereby reducing ROS levels and oxidative stress in the brains of APP/PS1 transgenic mice, which improves cognitive function in these AD models. *In vitro* experiments have also shown that anthocyanins mitigate neurotoxicity induced by amyloid *β* oligomers (AβO) through the PI3K/Akt/Nrf2 signaling pathway (44). In addition, anthocyanins-containing PEG-AuNPs (12 μg/g/day for 14 days) also modulated the p-PI3K/p-Akt/p-GSK3β pathway, thereby inhibiting the hyperphosphorylation of tau at serine 413 and 404 and apoptosis of neurons in the brains of mice injected with Aβ_1-42_ (45). Excessive neuroinflammation is directly related to the development of AD, and microglia are the main effectors of neuroinflammation (46). Supplementing with bilberry anthocyanins (20 mg/kg/day for 3 months) can activate astrocytes and microglia, and improve their phagocytic function of beta amyloid plaques in APP/PSEN1 mice (47). Activation of c-Jun N-terminal kinase (JNK) in the brain can stimulate microglia and increase the expression of proinflammatory cytokines, including tumor necrosis factor-alpha (TNF-*α*), interleukin-6 (IL-6), and monocyte chemoattractant protein-1 (MCP-1) (48), anthocyanin supplementation in LPS treated mice inhibited JNK activation and reduced the expression of nuclear factor kappa-B (NF-κB), TNF-α and interleukin-1beta (IL-1β). In addition, anthocyanins also reduced neuroinflammatory markers in Aβ_1-42_-induced mouse model by inhibiting the p-JNK/NF-κB/p-GSK3β pathway (49). High-fat diet is an important risk factor for inducing neurodegenerative diseases (50). Anthocyanin supplementation (4% blueberry diet for 5 months) was able to reverse some of the behavioral deficits in high-fat diet-induced mice, particularly object recognition memory (51). The neuroprotective effects of anthocyanins may be related to attenuated microglial activation and increased neuroplasticity (52). Anthocyanin supplementation (100 mg/kg/day for 20 weeks) could also further block oxidative stress by improving AMPK-mediated autophagy, restore brain-derived neurotrophic factor protein levels in the hippocampus of mice on a high-fat diet, and ultimately inhibit hippocampal cell apoptosis and ameliorate cognitive deficits (53). Anthocyanins (700 mg/kg/day for 20 weeks) can also alleviate high-fat diet-induced neuroinflammation by inhibiting extracellular signal-regulated kinases, JNK, p38, and NF-κB activation (54). In summary, both animal studies and randomized clinical trials demonstrate that anthocyanins enhance cognition and neuroprotection. The mechanisms underlying these neuroprotective benefits are linked to anthocyanins’ ability to reduce oxidative stress, inflammation, and apoptosis in the brain. To fully realize the neuroprotective effects, further research should determine the best dose and frequency of anthocyanins for human use (Table 2).

**Table 2:** Common dietary sources of anthocyanins and health outcomes associated with aging-related diseases.

Dietary	Effect	Suggested health outcomes
Cherry	Antioxidant; Lower blood pressure	↑Verbal fluency, motor, memory function, and lifespan (38, 64).
Blueberry	Regulate hormone levels; Lower blood pressure	↑Memory, brain neural activation and neuroprotective (39, 40, 51, 66); ↓Bone loss caused by ovariectomy (89, 90); ↑Vasodilation function (100, 101).
Black bean	Antioxidant; Pro-apoptotic	↑Memory functions (44); ↓BPH (128).
Mulberry	Antioxidant; Anti-apoptotic	↑Learning, motor and memory abilities (42); ↓Dopaminergic neuronal damage (58, 59); ↓Endothelial senescence (96).
Blackcurrant	Antioxidant; Anti-inflammatory; Lower blood pressure; Regulate hormone levels; Improved glycolipid metabolism	↑Neural response and emotional health (41, 57); ↑Cancellous bone mass (83); ↓Bone loss caused by ovariectomy (88); ↓Intraocular pressure (125); ↓Arterial stiffness (103); ↓Cardiometabolic risk (119); ↓Blood glucose and blood lipid (117).
Bilberry	Anti-inflammatory; Improved glycolipid metabolism	↑Cognitive function (47); ↑Visual function (126); ↓Cardiometabolic risk (119); ↓Blood glucose and blood lipid (117).
Purple sweet potato	Antioxidant; Anti-inflammatory	↑Memory function and neuroprotective (53, 54); ↓Development of atherosclerotic lesions (93).
Blackberry	Modulate gut microbiota composition	↓Neuroinflammation (63).
Black carrot	Antioxidant; Anti-apoptotic	↓Neurotoxicity (65).
Strawberry	Reduced astrocytosis	↑Grip strength and neuromuscular junction integrity (68).
Purple corn	Pro-apoptotic	↓BPH (129).
Grape skin	Regulate hormone levels	↓BPH (127).
Red Chinese cabbage	Anti-inflammatory	↓Risk of vascular inflammatory disease (94);
Black rice	Anti-inflammatory; Regulate intestinal flora; Regulatory bone turnover	↓Bone loss due to diabetes (85); ↑Intestine barrier integrity, ↓colorectal cancer cell proliferation (107).
Wheat	Enhanced autophagy	↑Antineoplastic activity (110).
Cranberry	Lower blood pressure	↓Risk of cardiovascular disease (102).
Raspberry	Lower blood pressure	↓Dementia patients’ blood pressure (104).
Purple rice	Anti-inflammatory	↓Articular cartilage degradation (73).
Maqui berry	Regulate hormone levels	↓Bone loss caused by ovariectomy (87).

BPH, benign prostatic hyperplasia; ↑, increase or enhance; ↓, reduce or inhibit.

#### 4.1.2 Anthocyanins and Parkinson’s disease

Aging significantly increases the risk of developing Parkinson’s disease (PD), with prevalence rising from age 50 to 80. The pathogenesis of PD is diverse, including *α*-synuclein misfolding and aggregation, oxidative stress, mitochondrial dysfunction, and neuroinflammation (55). Current treatments for PD are limited; common medications provide only symptom relief and often come with significant side effects. Human studies have shown that dietary anthocyanins can effectively reduce mortality risk and have a positive impact on the mood of patients with PD (56, 57). The main lesions in Parkinson’s disease are the midbrain substantia nigra (SN) and the striatum, accompanied by degeneration and death of dopaminergic neurons. 1-methyl-4-phenyl-1,2,3,6-tetrahydropyridine (MPTP) induces the death of specific dopaminergic neurons. Mulberry (*Morus alba* L.) extract (500 mg/kg/day for 15 days) can mitigate this cell death, reduce pro-apoptotic protein levels, and alleviate symptoms of Parkinson’s disease (58). A similar study also suggests that the mulberry (*Morus alba* L.) extract (250 mg/kg/day for 38 days) significantly inhibited the expression of Lewy body *α*-synuclein and ubiquitin, which are induced by MPTP (59). The injection of 6-hydroxydopamine (6-OHDA) leads to oxidative damage in neurons, which is associated with the death of neurons in Parkinson’s disease (60). Pelargonidin supplementation (20 mg/kg 1 day before and on the day of surgery) significantly increased the number of dopaminergic neurons in the substantia nigra, reduced lipid peroxidation levels, and improved motor function in rats that were injected with 6-OHDA (61). Some studies indicate that neurodegeneration in Parkinson’s disease (PD) is linked to gastrointestinal dysregulation. Additionally, anthocyanin supplementation exhibits neuroprotective effects in PD mice (10, 20, 40 mg/kg/day for 4 weeks) (62) and high-fat diet-induced obese rats (25 mg/kg/day for 17 weeks) (63) by modulation the composition and metabolism of gut microbiota. Moreover, other foods rich in anthocyanins, including sweet cherries (64), black carrot (65), blueberries and grape seed (66), may alleviate PD symptoms by providing antioxidant benefits, preventing cell death, and improving mitochondrial function. These studies indicate that anthocyanins could be a promising new element of treatment strategy for PD, requiring further investigation in clinical trials (Table 2).

#### 4.1.3 Anthocyanins and amyotrophic lateral sclerosis

Amyotrophic lateral sclerosis (ALS) is a severe neurodegenerative disease characterized by the progressive degeneration and death of motor neurons. While current research on anthocyanins in ALS remains limited, preliminary evidence from animal models suggests potential protective effects on ALS. For instance, anthocyanin-derived metabolites such as protocatechuic acid (100 mg/kg after onset until death, 1 time/day, 5 times/week) (67) and anthocyanin-enriched strawberry extract (2 mg/kg/day after 60 days of age until death) (68), were shown to attenuate spinal cord astrogliosis, inhibit motor neuron apoptosis, and preserve neuromuscular junction integrity in SOD1 mutant mice—a widely used ALS model. These interventions reportedly delayed disease progression, improved motor performance, and extended survival in preclinical settings. However, no recent studies have further explored anthocyanins’ therapeutic mechanisms or translational potential in ALS, nor have clinical trials investigated their efficacy in human patients. The scarcity of research highlights a critical gap in understanding how dietary polyphenols might intersect with ALS pathophysiology. Future studies should prioritize (1) validating these findings in additional ALS models (e.g., TDP-43 or C9orf72-related models), (2) elucidating gut-brain axis interactions, and (3) assessing bioavailability and dosing regimens for clinical translation (Table 2).

### 4.2 Anthocyanins and bone diseases

#### 4.2.1 Anthocyanins and osteoarthritis

Osteoarthritis (OA) is a chronic disease primarily affecting the elderly. A global study showed that there were about 300 million cases of hip and knee OA in 2017 (69). As the elderly population increases, osteoarthritis has emerged as a serious disease that affects their quality of life. The main pathological mechanism of OA is the degradation of the articular cartilage matrix, whose formation is related to cellular senescence, aging-related mitochondrial dysfunction, and oxidative stress (70). Additionally, both malvidin and pelargonidin can alleviate inflammation, cartilage degradation, and pain in OA by inhibiting the NF-κB pathway (71). Drugs for osteoarthritis (OA) can have several side effects, while nutritional health products are seen as an effective alternative for protecting and enhancing bone health (72). Studies have shown that anthocyanins improve OA symptoms by inhibiting inflammatory responses and the degradation of articular cartilage. For example, the anthocyanin in purple rice (6.25–50 μg/mL for 35 days) can reduce articular chondrocyte damage by inhibiting IL-1β-induced matrix metalloproteinase expression, which is closely related to the NF-κB and ERK/MAPK signaling pathways (73). Additionally, malvidin (74) and pelargonidin (75) can enhance the inflammatory response, reduce cartilage degradation, and alleviate pain in OA by inhibiting the NF-κB pathway. Research indicates that Sirtuin 6 improves chondrocyte aging and slows the progression of osteoarthritis (OA) (76). Cyanidin administration (50 mg/kg/day for 8 weeks), both *in vivo* and *in vitro*, enhances Sirt6 activity and inhibits the NF-κB signaling pathway. It also prevents IL-1β-induced degradation of the extracellular matrix (ECM) and reduces the inflammatory response in human OA chondrocytes. Additionally, it mitigates proteoglycan loss and cartilage damage caused by destabilization of the medial meniscus (DMM) in OA mice (77). Clinical studies indicate that consuming foods rich in anthocyanins can help balance immune markers in patients with osteoarthritis, thereby reinforcing the potential of anthocyanins as an additional therapeutic strategy (78). Therefore, anthocyanins can reduce OA symptoms and improve patients’ quality of life (Table 2).

#### 4.2.2 Anthocyanins and osteoporosis

Osteoporosis is a disease marked by low bone mass and changes in bone microstructure, leading to increased fragility and susceptibility to fractures, which adversely impacts patients’ quality of life (79). Advanced age is a major risk factor for chronic diseases. Hormonal imbalances that occur with age lead to dysfunction of osteoclasts and osteoblasts, oxidative stress, and chronic inflammation, all of which significantly contribute to the development of osteoporosis (80, 81). Anthocyanins are known for their anti-inflammatory, anti-oxidative, and anti-apoptotic effects. Studies suggest that anthocyanin-rich foods can improve bone remodeling biomarkers in middle-aged and elderly people, indicating their potential role in osteoporosis management (82). Sakaki et al. found that blackcurrant diet (a standard chow diet with 1% (w/w) anthocyanin for 4 months) improved cancellous bone mass loss in young mice by increasing glutathione peroxidase (GPX) activity in the humerus. However, this diet only modestly reduced TNF-*α* expression in older mice, with no significant effect on cancellous bone mass. This suggests that early administration of anthocyanins may help prevent age-related bone loss (83). Osteoporosis involves a gradual decline in osteoblasts and increased bone resorption by osteoclasts. Cyandin-3-glucoside plays a role in regulating osteoblast differentiation via the ERK1/2 signaling pathway (84). Osteoporosis related to diabetes is a systemic endocrine metabolic bone disease characterized by reduced bone density and destruction of bone microstructure. Studies show that anthocyanins in black rice extract (0.5, 1.0 and 2.0 g/kg/day for 8 weeks) can improve bone loss in diabetes rats by inhibiting bone turnover and bone marrow fat production, and up regulating the ratio of RUNX2 and OPG/RANKL in bone tissue of diabetes rats (85). Decreased estrogen levels are the primary cause of bone loss in postmenopausal women, with more than 30% of them affected by osteoporosis (86). Studies have demonstrated that supplements containing anthocyanins from blueberries, blackcurrants, or maqui berries can reduce bone loss induced by ovariectomy (87–90). This finding suggests that anthocyanins may help alleviate osteoporosis in postmenopausal women; however, the exact mechanism of action remains unclear and requires further investigation. Thus, anthocyanins, as natural bioactive compounds, may offer innovative strategies for preventing and treating osteoporosis (Table 2).

### 4.3 Anthocyanins and cardiovascular diseases

#### 4.3.1 Effects of anthocyanins on endothelial function

Aging is a complex biological process, and epidemiological studies prove that aging is an independent risk factor leading to the occurrence of cardiovascular diseases. As people age, the heart transitions from compensatory adaptation to maladaptation, resulting in cardiac hypertrophy, changes in left ventricular diastolic function and contractile reserve, increased arterial stiffness, and impaired endothelial function (1). Cardiac dysfunction due to aging can lead to various cardiovascular diseases, including atherosclerosis, hypertension, and dyslipidemia. Atherosclerosis is a chronic and progressive vascular disease that is a precursor of an ischemic heart attack. The initial stage of atherosclerotic lesion development involves the activation of endothelial cells. Activated endothelial cells release the inflammatory mediator MCP-1 into the bloodstream and express adhesion molecules (ICAM-1 and VCAM-1) to attract circulating monocytes and other immune cells to the site of oxidized low-density lipoprotein accumulation (91, 92). Oral administration of anthocyanins has been recognized as a therapeutic option for managing cardiovascular disease. Research indicates that purple sweet potato, red Chinese cabbage (93, 94), and protocatechuic acid (95) can reduce plasma VCAM-1 levels and inhibit the expression of adhesion molecules on arterial endothelial surfaces. Furthermore, daily intake of an extract high in Chinese cabbage anthocyanins (150 and 300 mg/kg/day for 12 weeks) can lower inflammatory cytokines and adhesion molecule levels, thus preventing plaque buildup in the arteries of hyperlipidemic mice (94). This suggests that anthocyanins suppress inflammation and alleviate the progression of atherosclerosis. Furthermore, Cyanidin-3-O-*β*-glucoside (100, 200 and 300 mg/kg for 8 weeks) enhances endothelial nitric oxide synthase phosphorylation and preserves nitric oxide availability, promoting endothelial cell migration and survival (96, 97). Cyanidin-3-O-β-glucoside (0.2% C3G for 6 weeks) also enhances the function of endothelial progenitor cells and promotes endothelial repair, thereby slowing atherosclerosis in apolipoprotein E-deficient mice (98). More importantly, anthocyanin metabolites enhance endothelial function by influencing the gut microbiota (99). These studies suggest that anthocyanins slow atherosclerosis progression by regulating vascular endothelial function. In conclusion, anthocyanins are crucial for cardiovascular health due to their antioxidant and anti-inflammatory properties, as well as their role in regulating endothelial cell function (Table 2).

#### 4.3.2 Anthocyanins and hypertension

Hypertension is a significant risk factor for cardiovascular diseases. Chronic hypertension increases the heart’s workload, requiring it to pump blood more forcefully. Over time, this can lead to cardiac hypertrophy and ultimately result in heart failure. In addition, hypertension damages vascular endothelial cells and promotes the formation of atherosclerosis, which can lead to coronary heart disease, strokes, and other serious conditions. Clinical pilot studies indicate that healthy elderly individuals aged 65 to 80, who consume 26 grams of freeze-dried wild blueberry powder (containing 302 mg of anthocyanins) daily for 12 weeks, experience significant increases in blood flow-mediated vasodilation and decreases in 24 h dynamic systolic blood pressure compared to the placebo group (100). Studies have also found that 5-week low-dose wild blueberry extract (222 mg of anthocyanins) significantly reduced systolic blood pressure in healthy elderly people (68–75 years old) (101). Moreover, a 6-week regimen of 85 mg of cranberry extract (25% anthocyanins) per day significantly lowered both systolic and diastolic blood pressure in patients with myocardial infarction (102). Short-term (28 days) ingestion of 300 mg New Zealand blackcurrant extract capsules (35% blackcurrant extract) reduced arterial stiffness and blood pressure in elderly individuals with an average age of 73.3 years (103). These studies indicate that anthocyanins can lower blood pressure. Growing evidence suggests that anthocyanin’s anti-hypertensive effects primarily stem from its antioxidant, anti-inflammatory, and ACE inhibitory properties, along with its ability to inhibit the growth of vascular endothelial cells. However, the intake of anthocyanin-rich blood orange juice (50 mg/500 mL) for 4 weeks also had no effect on blood pressure in healthy people (25–84 years old) (104), but it could significantly reduce blood pressure in patients with dementia (104). Therefore, the antihypertensive effects of anthocyanins depend on both the dosage and the duration of administration, and additional clinical trials are needed to determine the ideal nutritional intake and specific mechanisms, which will help create a stronger scientific basis for the prevention and treatment of cardiovascular diseases (Table 2).

### 4.4 Anthocyanins and cancer

Aging is a key risk factor for both the onset and progression of cancer, which is a leading cause of the rising mortality rate globally (105). Several studies have confirmed that anthocyanins possess anti-cancer properties. For example, it has been shown that anthocyanins (200 mg/kg) are able to significantly inhibit the growth of colorectal cancer cells, and to promote the apoptosis of cancer cells by regulating the PI3K/AKT signaling pathway (106). Anthocyanin can also further activate the aryl hydrocarbon receptor pathway by regulating intestinal flora, improve the intestinal barrier function, reduce inflammatory, and inhibit the proliferation and cell cycle of colorectal cancer cells (107). In addition, anthocyanins can slow tumor development by inhibiting tumor-associated inflammatory responses and reducing pro-inflammatory factors in the tumor microenvironment (20). In breast cancer and prostate cancer studies, anthocyanins have inhibited the growth of cancer cells by regulating the cell cycle and inducing apoptosis, thus showing a good preventive effect (108, 109). The latest studies show that anthocyanin-rich cereal diets (anthocyanin content 140 mM/g for 4.5 months) enhance autophagy by reducing M1 macrophage markers in tumors and promoting the expression of M2 macrophage markers, thereby exerting antitumor effects in Lewis lung cancer mice (110). Anthocyanins diet (0.5% CAN for 15 weeks) can also reduce lipid deposition in cancer cells by regulating the AMPK/mTOR signaling pathway, thereby inhibiting the development of urethane-induced lung cancer in C57BL/6 J mice (111). While multiple studies have confirmed the anti-cancer effects of anthocyanins, further research is needed to determine their effective dosage and long-term clinical effects. Furthermore, the current study has focused on the relationship between anthocyanins intake and cancer risk, and some epidemiological studies showing that a diet rich in anthocyanins may be associated with reduced risk of some cancers (112). However, more randomized controlled trials are still needed to validate the specific mechanism of action of anthocyanins and its potential use in cancer prevention (Table 2).

### 4.5 Anthocyanins and metabolic syndrome

Aging is a major risk factor for developing metabolic syndrome, a complex condition characterized by symptoms like obesity, glucose intolerance, insulin resistance, dyslipidemia, and hypertension (113). These symptoms significantly increase the risk of cardiovascular disease and diabetes mellitus. Compared with healthy individuals, the proliferation of harmful bacterial flora in the gut of patients with metabolic syndrome is increased, and the beneficial bacterial flora is inhibited (114). Research shows that anthocyanin metabolites promote the growth of beneficial gut flora, improving intestinal health and metabolic function (115). Chronic inflammation is a hallmark of metabolic syndrome, and anthocyanins (320 mg/day for 4 weeks) can lower systemic inflammation by inhibiting proinflammatory factors like TNF-*α* and IL-6, thus alleviating metabolic syndrome symptoms (116, 117). Anthocyanins can also ameliorate the development of metabolic syndrome by improving the hypertrophy and inflammatory status of adipose tissue by regulating the leptin signaling pathway (118). These studies suggest that anthocyanins alleviate key features of metabolic syndrome by regulating gut microbiota, reducing chronic inflammation, and modulating leptin signaling pathways. In addition, anthocyanins play an important role in regulating lipid metabolism. Studies have shown that anthocyanins (640 mg/day for 4 weeks) can reduce the levels of low-density lipoprotein cholesterol (LDL-C) and triglyceride (TG) levels in serum, while increasing the level of high-density lipoprotein cholesterol (HDL-C), thus improving abnormal lipid metabolism (119, 120). Anthocyanins (320 mg/day for 4 weeks) effectively improve insulin resistance by activating AMPK and PPAR-*γ* signaling pathways, enhancing cell sensitivity to insulin (99, 117). Oxidative stress is considered a key factor in the development of metabolic syndrome, and anthocyanins effectively reduce oxidative damage by scavenging free radicals and boosting the activity of antioxidant enzymes (116, 121). These researches indicate that anthocyanins mitigate metabolic disturbances associated with metabolic syndrome by regulating lipid metabolism, insulin sensitivity, and antioxidative stress. Although these studies provide a rationale for anthocyanins as a natural drug for potential antimetabolic syndrome, future studies should investigate the potential benefits and optimal dosing of anthocyanins in clinical applications (Table 2).

### 4.6 Anthocyanins and other diseases

Glaucoma is a chronic, progressive optic nerve disease that is a leading cause of irreversible blindness worldwide (122). The likelihood of developing glaucoma and other common eye diseases, like cataracts and macular degeneration, rises with age (123, 124). Studies have shown that black currant anthocyanins (50 mg/day for 4 weeks) are effective in lowering intraocular pressure in both healthy individuals and glaucoma patients (125). Additionally, bilberry anthocyanins (120 mg/day for 24.32 ± 10.34 months) can improve visual function in patients with normal-tension glaucoma (126). However, the specific mechanism of action has not been reported in the literature and requires further investigation. Benign prostatic hyperplasia (BPH) is a common chronic disease of the urinary system among elderly men. An imbalance of androgens in older men is one of the main causes of BPH. Dihydrotestosterone (DHT) and converted testosterone by 5-*α* reductase type 2 (5AR2), binding with androgen receptor (AR), affect prostate proliferation and growth. In BPH, androgen signaling boosts the levels of prostate-specific antigen (PSA) and certain cytokines, like proliferating cell nuclear antigen (PCNA) and cyclin D1. Research has demonstrated that polymerized anthocyanin (PA) reduces the expression of proteins related to androgen signaling, including 5AR2, AR, and PSA in LNCaP cell lines. Oral administration of PA (100 mg/kg/day for 4 weeks) can reduce the expression levels of AR, 5ar2, PSA, PCNA, cyclin D1, Bcl-2 in prostate tissue and serum DHT level, ultimately improving prostate weight in rats with BPH (127). Similarly, Jang et al. demonstrated that a 4-week black soybean (40, 80, and 160 mg/kg for 4 weeks) anthocyanin intervention effectively reduced prostate volume in benign prostatic hyperplasia (BPH) rats (128). Meanwhile, purple corn extract enhanced (10, and 50 mg/kg/day for 4 weeks) pro-apoptotic gene expression by inhibiting androgen and AR signaling markers and regulating the PI3K/AKT signaling cascade, resulting in reduced prostate hypertrophy weight (129). These findings suggest that anthocyanin may be a promising natural treatment for BPH (Table 2).

## 5 Conclusion

**Figure 1:**
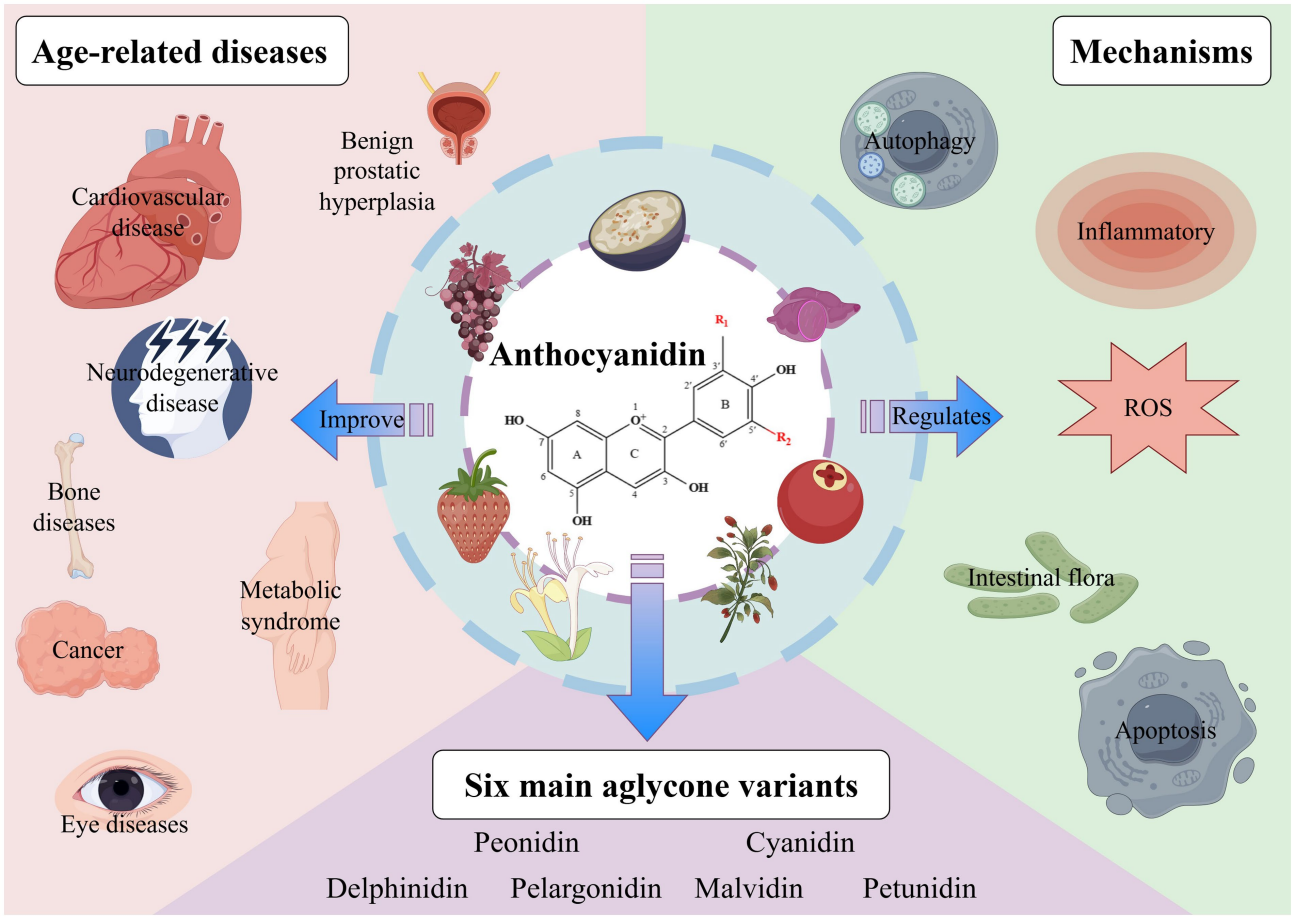
The chemical properties, biological functions, and ability to improve age-related diseases of anthocyanins. ROS, reactive oxygen species. Created with Figdraw.com.

Anthocyanins, as plant-derived bioactive compounds, hold significant potential to extend lifespan and combat age-related diseases through pleiotropic mechanisms such as autophagy activation, oxidative stress reduction, and promoting intestinal health. Preclinical evidence supports their therapeutic benefits in neurodegenerative disorders, osteoporosis, cancer, cardiovascular diseases, and other aging-associated conditions, mediated by antioxidant, anti-inflammatory, and metabolic regulatory properties (Figure 1). However, critical knowledge gaps persist in translating these findings to elderly populations: (1) Existing studies predominantly rely on animal models or young/middle-aged cohorts, with minimal data on long-term efficacy and safety in frail older adults (≥75 years) exhibiting multimorbidity or polypharmacy. (2) Age-related declines in gastrointestinal absorption, hepatic metabolism, and renal excretion may alter anthocyanin pharmacokinetics, yet no studies have systematically addressed this. (3) The impact of genetic polymorphisms (e.g., GST enzymes), sex hormones, and baseline microbiota diversity on anthocyanin effects remains unexplored in aging contexts.
